# Supplementary figures and images for: Live-Cell Microscopy Reveals That Human T Cells Primarily Respond Chemokinetically Within a CCL19 Gradient That Induces Chemotaxis in Dendritic Cells
Source: Front Immunol. 2021 Mar 26;12:628090. doi: 10.3389/fimmu.2021.628090 (PMC8033042; doi:10.3389/fimmu.2021.628090)

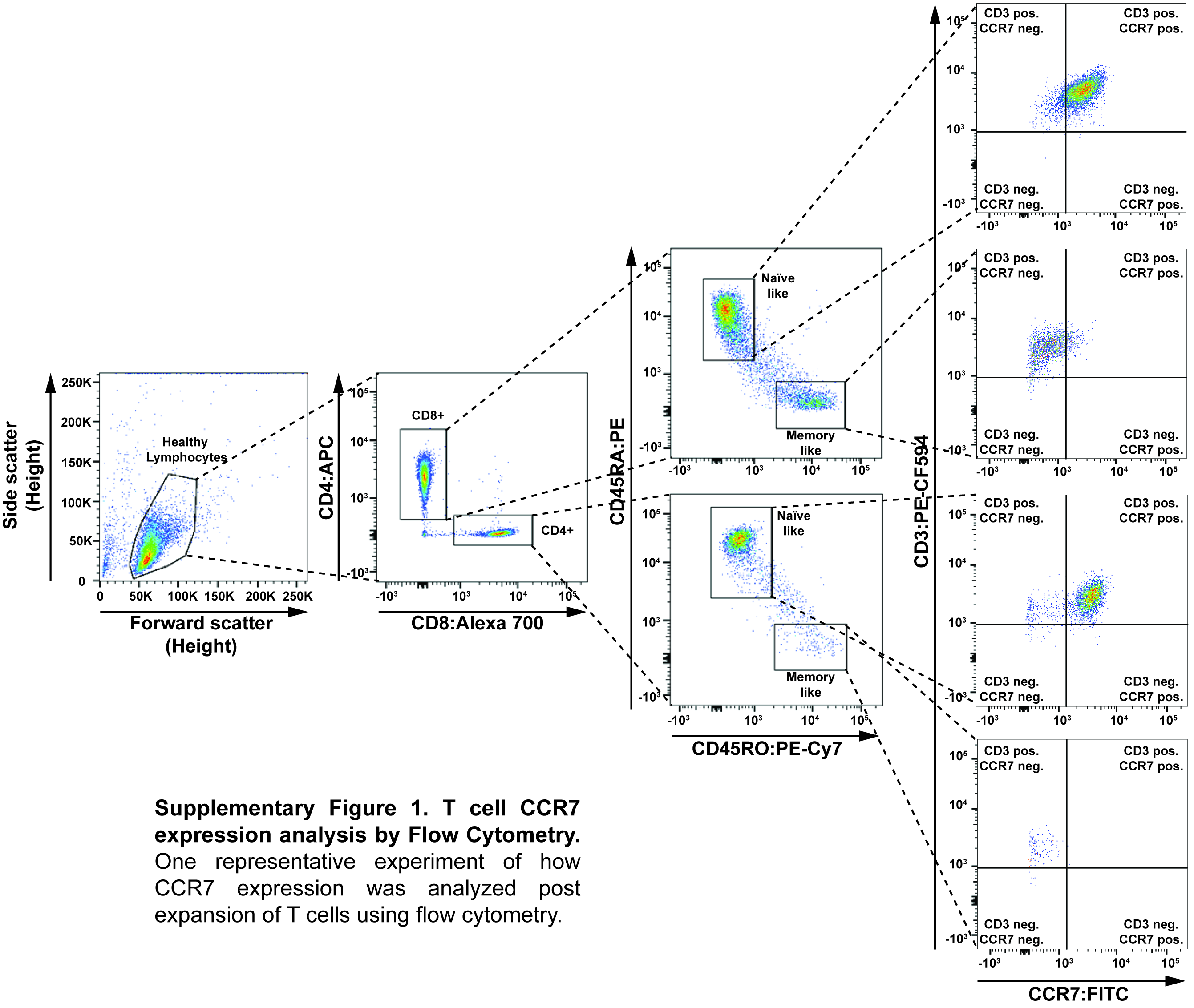

Supplement: Supplementary file 1 [file Image_1.tif]

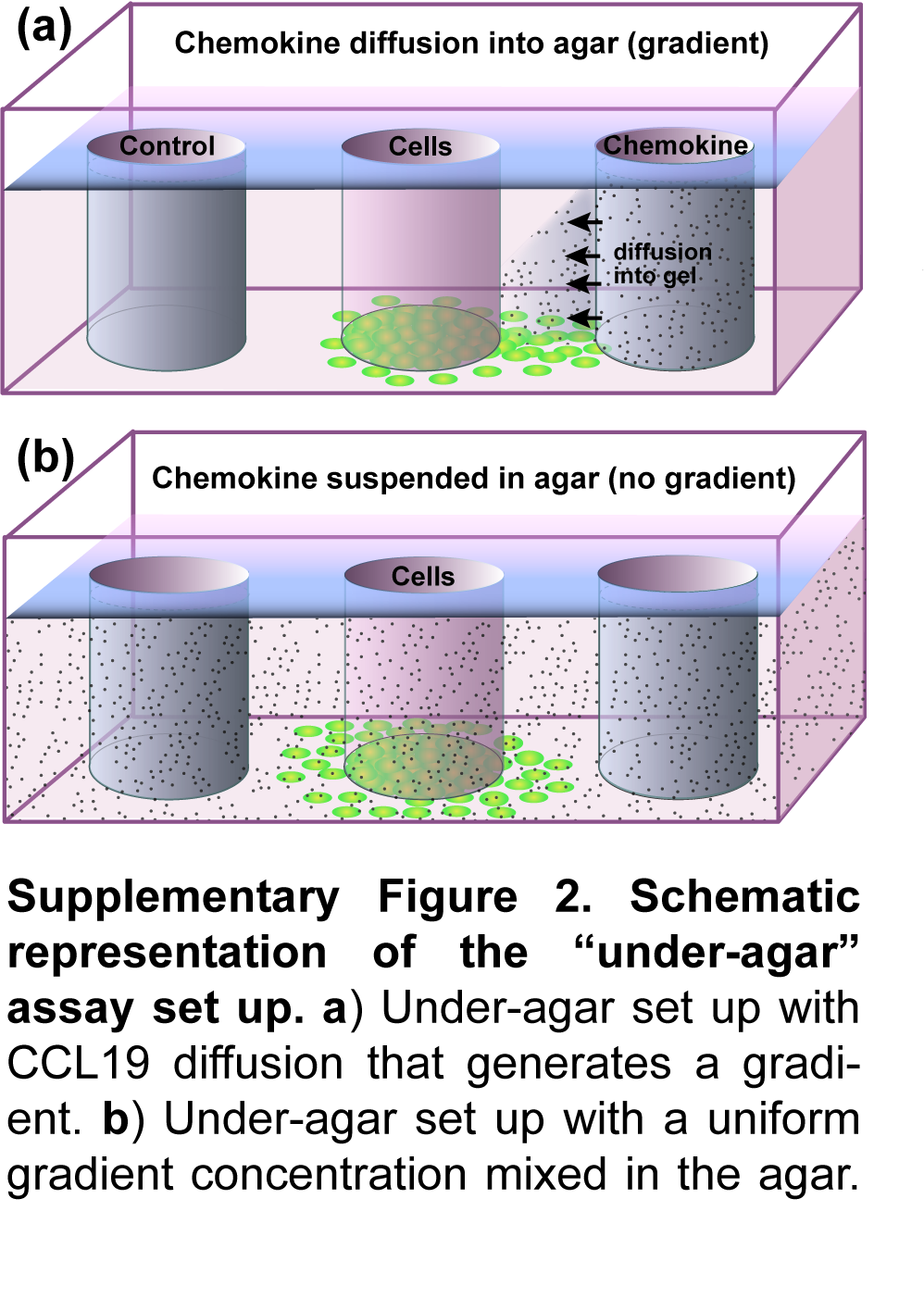

Supplement: Supplementary file 2 [file Image_2.tif]
