# Supplementary material for: Live-Cell Microscopy Reveals That Human T Cells Primarily Respond Chemokinetically Within a CCL19 Gradient That Induces Chemotaxis in Dendritic Cells
Source: Front Immunol. 2021 Mar 26;12:628090. doi: 10.3389/fimmu.2021.628090 (PMC8033042; doi:10.3389/fimmu.2021.628090)
Supplement: Supplementary file 10 [file Table_1.docx]

Supplementary Material

**Supplementary Figure 1. T cell CCR7 expression analysis by Flow Cytometry.** One representative experiment of how CCR7 expression was analyzed post expansion of T cells using flow cytometry.

**Supplementary Figure 2. Schematic representation of the “under-agar” assay set up.** a) Under-agar set up with CCL19 diffusion that generates a gradient. b) Under-agar set up with a uniform gradient concentration mixed in the agar.

**Supplementary video 1. Gradient formation and mDCs response to CCL19 in the under-agar assay.** 10 kDa fluorescent dextrans (yellow) were added at the same time as 100 ng CCL19 to analyze the diffusion and the migration of mDCs (cyan) in the under-agar assay in real-time.

**Supplementary video 2. mDCs migrating without chemokine.** Time-lapse showing the migration response of mDCs up to 6 hours after being added the under-agar assay without the presence of chemokine.

**Supplementary video 3. mDCs migrating in a uniform CCL19 concentration.** Time-lapse showing the migration response of mDCs up to 6 hours after being added the under-agar assay containing a uniform 100 ng mL^-1^ CCL19 concentration.

**Supplementary video 4. T cells migrating to a CCL19 gradient.** Time-lapse showing the migration response of T cells up to 6 hours after 100 ng CCL19 was added to the under-agar assay.

**Supplementary video 5. T cells migrating in a uniform CCL19 concentration.** Time-lapse showing the migration response of mDCs up to 6 hours after being added the under-agar assay containing a uniform 100 ng mL^-1^ CCL19 concentration.

**Supplementary video 6. mDCs and T cells migrating in a co-culture to a CCL19 gradient.** Time-lapse showing the migration response of mDCs (red) and T cells (green) in co-culture up to 6 hours after 100 ng CCL19 was added to the under-agar assay.

**Supplementary video 7. mDCs and T cells migrating in a co-culture to a uniform CCL19 concentration.** Time-lapse showing the migration response of mDCs (red) and T cells (green) in co-culture up to 6 hours after being added the under-agar assay containing a uniform 100 ng mL^-1^ CCL19 concentration.
